# Synthesis of Novel Fluorene Bisamide Derivatives via Ugi Reaction and Evaluation their Biological Activity against *Mycobacterium species*

**Published:** 2017

**Authors:** Ali Hossein Rezayan, Safoura Hariri, Parisa Azerang, Ghazaleh Ghavami, Isabel Portugal, Soroush Sardari

**Affiliations:** a *Department of Life Science Engineering, Faculty of New Sciences and Technologies, University of Tehran, ,Tehran, Iran.*; b *Drug Design and Bioinformatics Unit, Department of Medical Biotechnology, Biotechnology Research Center, Pasteur Institute of Iran, Tehran, Iran.*; c *iMed.ULisboa, Instituto de Investigação do Medicamento, Faculdade de Farmácia da Universidade de Lisboa, Av. das Forcas Armadas 1649-019 Lisboa, Portugal.*

**Keywords:** multi-component reaction, Ugi reaction, *Mycobacterium bovis*, fluorene bisamide derivatives, brine shrimp toxicity study

## Abstract

A series of new fluorene bisamide derivatives were synthesized through multi-component Ugi reaction and tested for their *in-vitro* anti-mycobacterial activity. The structures of the products 5a-w were deduced from their IR, 1H NMR, and 13C NMR spectra. Elemental analyses (CHN) for novel compounds (5a, 5d, 5f, 5h, 5k, 5l, 5p, 5s, 5t, 5v, 5w) was done. These compounds were evaluated as anti-bacterial agents against *Mycobacterium bovis *and *M. tuberculosis*, while their activity expressed as the minimum inhibitory concentration (MIC) in μg/mL. Among the twenty-three synthesized compounds, 5a was found to be the most active compound *in vitro* with MIC of 1.95 μg/mL against *Mycobactrium bovis *and compound 5k showed greatest potency against sensitive and resistant strains of *M.* *tuberculosis* (H37Rv, IHMT149/09, HPV115/08, and HPV65/08).

## Introduction

Tuberculosis (TB) is one of the deadly infectious diseases caused by *Mycobacterium* spp. mainly *Mycobacterium tuberculosis*. More than 2 billion humans (>30%) are infected by *Mycobacterium tuberculosis*, the causing organism of tuberculosis(1-5). *Bacillus Calmette-Guerin* (BCG) is an attenuated strain of *Mycobacterium bovis*, a non-virulent tubercle bacillus very closely related to *M. tuberculosis* (6, 7). Therefore, *M. bovis* is simpler to use and in less strict biosafety regulations in the lab, hence, it can be used in bioassay instead of *M. tuberculosis*.

The World Health Organization (WHO) *Fact Sheet* on TB estimates that between 2000 and 2020, nearly one billion people will get sick and 35 million will die from TB (8). Serious challenges associated with the rising epidemic are multidrug-resistance and the growing number of people co-infected with *M. tuberculosis *and human immunodeficiency virus (HIV) ( 9-11).

Hence, it is clear that there is an urgent need to develop novel anti-TB drugs with improved properties such as enhanced activity against multidrug-resistance, reduced toxicity, shortened duration of therapy, rapid mycobactericidial mechanism of action, ability to penetrate host cells, and exert anti-mycobacterial effect in the intracellular environment. There are various sources for providing molecules with the desired profile of biologic activity, among which natural products and synthetic reactions are two important ones. Although many natural products present the scaffolds with different applications, the synthetic routes have the advantage of diversity of variation in derivative functionalization. 

Multi-component reactions (MCRs) are special types of synthetically useful organic reactions in which three or more different starting materials react to give a final product in a one-pot protocol. Compared to conventional multistep organic syntheses, MCRs are advantageous owing to their greater atom efficiency, accessibility to large numbers of compounds and complex molecules, wide structural diversity and simplicity of their one-pot procedures making them amenable to combinatorial synthesis. 

Major applications of MCRs described until today arise from the area of drug discovery. Potentially, the ease of performance, the time-saving aspect, the versatility and diversity of scaffolds, and the very large chemical space will attract chemists in pharmaceutical companies to use MCRs for their projects. Recently, the pharmaceutical industries have focused more and more on diversity oriented combinatorial libraries (12-16). Among the known multi-component reactions to date, the most valuable reactions are those based on isocyanides. One such reaction is the Ugi 4-component condensation (4CC) reaction combining an amine, aldehyde (or ketone), carboxylic acid and isocyanide in a single-stage reaction to afford α-acylamino amides (17, 18).

Fluorenes are a useful class of compounds with high utility as building blocks for advanced materials with unique electrical and optical propertie (19-21), and also the fluorene unit frequently shows up in bioactive molecules.^22^ Therefore, in continuation of our research on the development of synthetic methods in heterocyclic chemistry via isocyanide based-multi-component reactions (23, 24) and also our drug discovery program (25-27) here we report synthesis of fluorene bisamide derivatives and evaluation of their anti-tuberculosis activity.

## Experimental

*General*

Melting points were measured on an Electrothermal 9100 apparatus and are uncorrected. Infrared spectra were determined with a Perkin-Elmer 843 spectrometer. Proton nuclear magnetic resonance (^1^H NMR) spectra and carbon nuclear magnetic resonance (^13^C NMR) spectra were determined on a Bruker Avance DRX 500 MHz spectrometer and chemical shifts are reported as δ (ppm) in CDCl_3 _and DMSO solution (0.05% v/v TMS). The chemicals used in this work were purchased from Merck, Fluka and Sigma-Aldrich Chemical Companies.

*General procedure for preparation of products 5a-w*

To a magnetically stirred solution of benzaldehyde derivatives (1 mmol) in methanol (5 mL) was added 2-fluoreneamine (1 mmol) and the reaction mixture stirred at room temperature until the imine was formed. After formation of imine, carboxylic acids (1 mmol) and cyclohexyl isocyanide (1.2 mmol) were added to reaction mixture in 0 ºC in ice bath and then reactions were continued in ambient temperature. After completion of the reaction, as indicated by TLC (ethyl acetate/n-hexane, 2:1), the precipitate was cooled at 0-5°C in an ice bath and was washed with cold hexane several times and then washed with freshly methanol and dried on the vacuum evaporator. Finally, the separated solid was filtered and purified by re-crystallization from ethanol to afford analytically pure product 5a-w.

2.2.1.N-[(cyclohexylcarbamoyl)(3-fluorophenyl)methyl]-N-(9H-fluoren-2-yl)prop-2-ynamide (5a). White solid; yield 75%, mp 199-200 °C; FT-IR (KBr): 1638, 2100, 2858, 2926, 3061, 3281 cm^-1^. ^1^H NMR (500 MHz,CDCl_3_): δ = 1.13 -2.02 (m, 10H), 2.85 (s, 1H), 3.81-3.93 (m, 3H), 5.72 (bs, 1H), 6.03 (s, 1H), 7.06-7.13 (m, 3H), 7.01-7.43 (m, 7H), 7.58 (d, ^3^*J*_HH _=7.4 Hz, 1H), 7.64 (d, ^3^*J*_HH _=8.0 Hz, 1H), 7.78 (d, ^3^*J*_HH _=7.5 Hz,1H).^ 13^C NMR (125 MHz, CDCl_3_): δ = 24.7, 24.8, 25.4, 32.7, 36.7, 48.9, 64.6, 76.0, 80.9, 115.7, 117.2, 119.6, 120.2, 125.1, 125.9, 126.9, 127.2, 129.2, 129.9, 130.0, 136.0, 137.2, 140.6, 142.1, 143.6, 143.7, 153.9, 162.2(d, ^1^*J*_CF_ = 244.5 Hz),167.2. Anal. Calcd for C_30_H_27_FN_2_O_2_: C, 77.23; H, 5.83; N, 6.00, Found: C, 77.44; H, 5.73; N, 5.92.

**Table 1:** The structure of fluorene bisamide derivatives and their MIC (µg/mL) against M. bovis BCG in micro broth dilution method assay

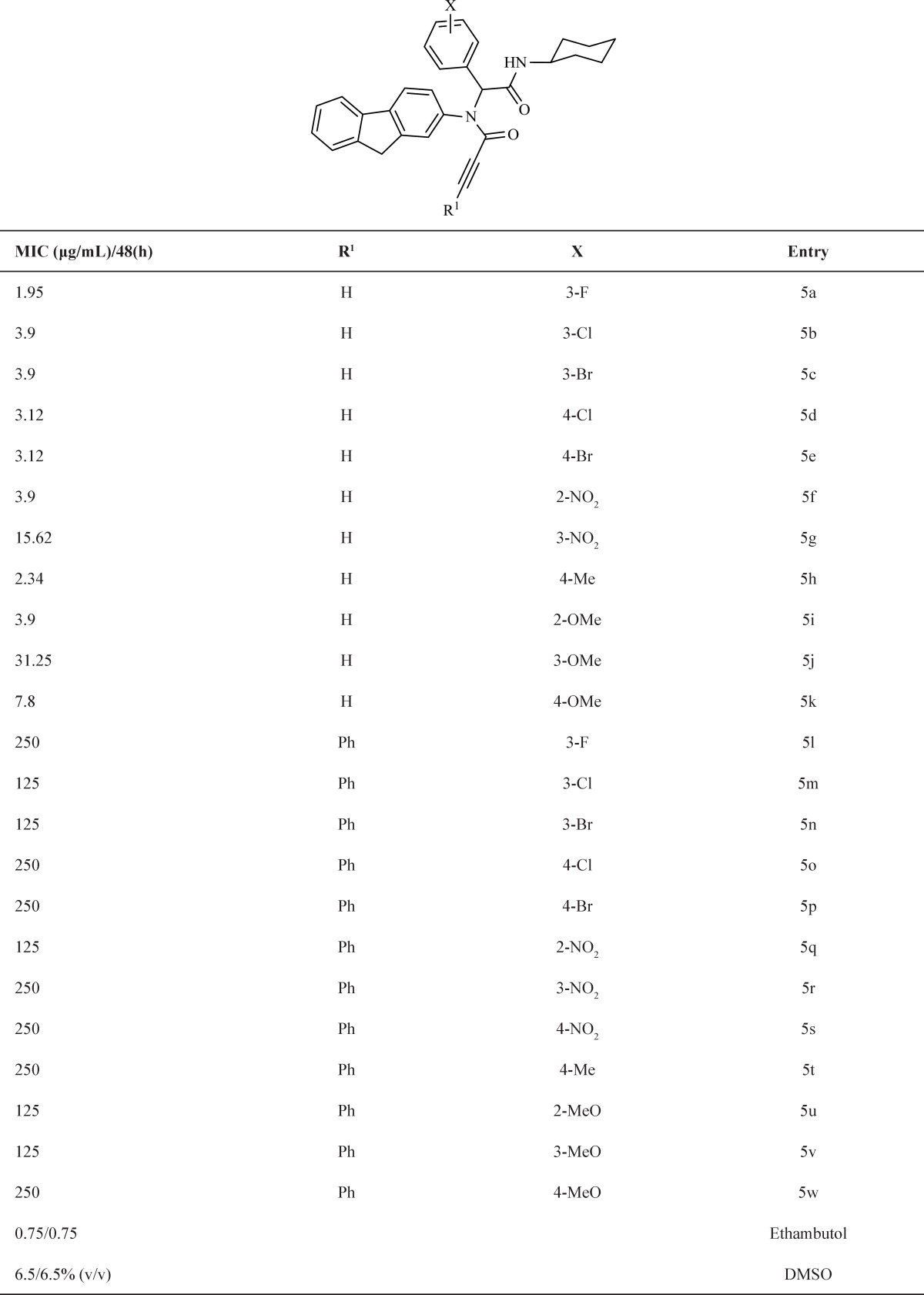

**Scheme 1:**
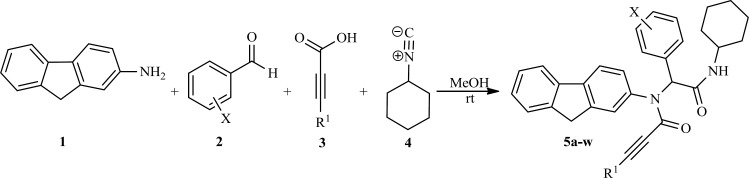
Synthesis of fluorene bisamide derivatives examined for anti-mycobacterial activity in this study

**Table 2.** Brine shrimp lethality bioassay

**Compound**	**LD**_50 _ **(µg/mL)** 1	**MIC (µg/mL)**					**SI** 4
		***M. bovis*** ** (1173P2)** 2	***M. tuberculosis*** 3				
			**H37Rv**	**IHMT295-08**	**HPV115-08**	**HPV65-08**	
5a	39.69	1.95	500	31.25	ND^5^	ND	20.35
5h	102.7	2.34	500	15.63	ND	ND	43.88
5k	6.063	7.8	1	1	1	ND	0.77
Galic acid	23.84 µg/mL on *A. salina*						
Ethambutol	7.81 µg/mL against * M. bovis*						
Isoniazid	<1.9 µg/mL for *M.* *bovis* **-** 0.06 and 40 µg/mL against sensitive and resistant strains of * M. tuberculosis*						
DMSO	1.25% v/v						

1 For * Artemia* lethality bioassay, the LD_50_s with 95% confidence intervals were estimated by GraphPad Prism 5.0 (2007) based on analytical method non-linear regression

2 MICs were estimated after 24 h based on broth microdilution method

3 MICs were estimated after 24 h based on broth microdilution method against H37Rv (Pansusceptible Reference Laboratory strain), IHMT149/09 (Clinical XDR-TB strain), HPV115/08 (Clinical XDR-TB strain), and HPV65/08 (Pansusceptible clinical strain), this part of assay were performed at Department of Microbiology, Faculdade de Farma´cia da Universidade de Lisboa, Lisbon, Portugal

4 The selectivity index (SI)s were determined by dividing LD_50 _to MIC (*M. bovis*)

5 not determined since positive control failed to grow

2.2.2.N-[(3-chlorophenyl)(cyclohexylcarbamoyl)methyl]-N-(9H-fluoren-2-yl)prop-2-ynamide (5b).White solid; yield 50%, mp 208-210 °C; FT-IR (KBr): 1677, 2111, 2855 , 2930, 3061, 3281 cm^-1^.^1^H NMR (500 MHz, CDCl_3_): δ= 1.05 -2.00 (m, 10H), 2.82 (s, 1H), 3.85 (m, 3H), 5.72 (bs, 1H), 6.01 (s, 1H), 7.06 -7.13 (m, 3H), 7.21-7.44(m, 4H), 7.55 (d, ^3^*J*_HH_ =7.4 Hz, 1H), 7.6 (d, ^3^*J*_HH_ =8.0 Hz, 1H), 7.75 (d, ^3^*J*_HH_ =7.5 Hz,1H). ^13^C NMR (125 MHz, CDCl_3_): δ = 24.6, 25.4, 32.7, 32.8, 36.7, 48.9, 64.5, 76.0, 80.9, 119.6, 120.2, 125.1, 126.8, 127.2, 128.3, 128.9, 129.2, 129.6, 130.4, 134.2, 135.6, 137.1, 140.6, 142.0, 143.6, 143.7, 153.9, 167.1. 

2.2.3.N-[(3-bromophenyl)(cyclohexylcarbamoyl) methyl]-N-(9H-fluoren-2-yl)-prop-2-ynamide (5c).White solid, yield 90%; mp 228-230 °C; FT-IR (KBr): 1678, 2112, 2855, 2930, 3270, 3283 cm ^-1^. ^1^H NMR (500 MHz, CDCl_3_): δ= 1.06 -2.01 (m, 10H), 2.82 (s, 1H), 3.79 -3.90 (m, 3H,), 5.70 (bs, 1H), 6.00 (s, 1H), 7.03 -7.11 (m, 3H), 7.32 -7.43 (m, 5H), 7.55 (d, ^3^*J*_HH_=7.3 Hz, 1H), 7.62(d, ^3^*J*_HH_ =8.0 Hz, 1H), 7.76 (d, ^3^*J*_HH_ =7.5 Hz, 1H). ^13^C NMR (125 MHz, CDCl_3_): δ = 24.6, 25.4, 32.71, 36.7, 48.9, 64.4, 76.0, 80.9, 119.6, 120.2, 122.3, 125.1, 126.8, 127.2, 127.3, 128.8, 129.32, 129.9, 131.8, 133.4, 135.9, 137.1, 140.6, 142.1, 143.6, 143.7, 153.9, 167.1.

2.2.4.N-[(4-chlorophenyl) ( cyclohexylcarbamoyl) methyl]-N-(9H-fluoren-2-yl)- prop-2-ynamide (5d).White solid; yield 55%; mp 196-197 °C; FT-IR (KBr): 1655, 2107, 2853, 2932, 3069, 3256, 3275 cm ^-1^.^1^H NMR (500 MHz, CDCl_3_): δ =1.05 -2.00(m, 10H), 2.82(s, 1H), 3.78-3.89(m, 3H), 5.67(bs,1H), 6.03(s, 1H), 7.4 -7.13 (m, 8H), 7.55(d, ^3^*J*_HH_= 7.4), 7.61(d, ^3^*J*_HH_= 7.9, 1H), 7.76(d, ^3^*J*_HH_= 7.5,1H).^13^C NMR (125 MHz, CDCl_3_): δ =24.6, 24.7, 25.3, 32.7, 32.8, 36.7, 48.9, 64.1, 76.0, 80.9, 119.6, 120.2, 125.1, 126.9, 127.2, 127.3, 128.7, 129.2, 129.3, 131.7, 132.1, 134.8, 136.8, 137.1, 140.6, 142.1, 143.5, 143.6, 153.9, 167.3. Anal. Calcd for C_30_H_27_ClN_2_O_2_: C, 74.60; H, 5.63; N, 5.80, Found: C, 74.81; H, 5.53; N, 5.72.

2.2.5.N-[(4-bromophenyl)(cyclohexylcarbamoyl) methyl]-N-(9H-fluoren-2-yl)- prop-2- ynamide(5e).White solid; yield 55% ; mp 199-202 °C; FT-IR (KBr): 1655, 2107, 2853, 2931, 3069, 3279 cm ^-1^.^1^H NMR (500 MHz, CDCl_3_): δ=1.06-1.99 (m, 10H), 2.82 (s, 1H, C≡CH), 3.78-3.89(m, 3H), 5.70(bs,1H), 6.02 (s, 1H), 7.07-7.76 (m, 11H),.^13^C NMR (125 MHz, CDCl_3_): δ = 24.7, 24.8, 25.4, 32.7, 32.8, 36.7, 48.9, 64.2, 76.0, 80.9, 119.6, 120.2, 123.1, 125.1, 126.9, 127.2, 129.3, 131.6, 132.0, 132.7, 137.1, 140.6, 142.1, 143.6, 143.7, 153.9, 167.2. 

2.2.6.N-[(cyclohexylcarbamoyl)(2-nitrophenyl)methyl]-N-(9H-fluoren-2-yl)-prop-2-ynamide (5f).White solid, yield 50 %; m.p. 179-180 °C; FT-IR (KBr): 1682, 2109, 2853, 2930, 3074(C-H Ph), 3299cm ^-1^.^1^H NMR (500 MHz, CDCl_3_): δ = 1.09-1.99(m, 10H),2.83(s, 1H), 3.75-3.85(m, 3H), 5.78(bs,1H), 6.56(s, 1H),7.31- 7.58(m, 7H), 7.71-7.91(m, 4H).^13^C NMR (125 MHz, CDCl_3_): δ =24.7, 24.8, 25.4, 32.6, 32.7, 36.7, 49.2, 60.1, 75.9, 80.8, 117.8, 119.6, 120.1, 124.6, 125.1, 126.9, 127.2, 128.1, 128.9, 129.7, 132.2, 132.7, 136.9, 140.5, 142.1, 143.6, 143.6, 149.7, 153.7, 166.9. Anal. Calcd for C_30_H_26_ClN_3_O_4_: C, 68.24; H, 4.96; N, 7.96, Found: C, 67.74; H, 5.05; N, 7.88.

2.2.7.N-[(cyclohexylcarbamoyl)(3- nitrophenyl)methyl]-N-(9H-fluoren-2-yl)- prop-2-ynamide (5g).White solid; yield 45%; mp 210-211 °C; FT-IR (KBr): 1647, 2112, 2853, 2928, 3089, 3302 cm ^-1^.^1^H NMR (500 MHz, CDCl_3_): δ =1.15- 2.05(m, 10H), 2.86 (s, 1H), 3.78-3.90(m, 3H,), 5.90(bs,1H), 6.14(s, 1H), 7.32-7.62(m, 7H), 7.74-8.18 (m,4H).^13^C NMR (125 MHz, CDCl_3_): δ = 24.7, 25.4, 29.6, 32.8, , 36.7, 49.1, 63.9, 75.7, 81.4, 119.8, 120.3, 123.6, 125.1, 125.4, 126.9, 127.1, 127.4, 129.1, 129.2, 135.5, 136.3, 136.5, 140.3, 142.4, 143.6, 143.9, 147.9, 154.1, 166.8.

2.2.8.N-[(cyclohexylcarbamoyl)(4-methylphenyl)methyl]-N-(9H-fluoren-2-yl)-prop-2-ynamide (5h). White solid; yield 80 %; m.p. 225 °C; FT-IR (KBr): 1683, 2108, 2853, 2924, 3054, 3275, 3319 cm ^-1^.^1^H NMR (500 MHz, CDCl_3_): δ = 1.12-1.98(m, 10H), 2.26 (s, 3H), 2.8 (s, 1H), 3.76-3.92 (m, 3H), 5.50(bs,1H), 6.02 (s, 1H), 6.99 -7.12 (m, 5H), 7.23-7.48 (m, 3H), 7.54 (d, ^3^*J*_HH_=7.45, 1H), 7.58 (d, ^3^*J*_HH_=7.95, 1H), 7.74 (d, ^3^*J*_HH_= 7.5, 1H).^13^C NMR (125 MHz, CDCl_3_): δ =21.1, 24.7, 24.8, 25.4, 29.6, 32.7, 36.7, 48.8, 64.9, 76.2, 80.4, 119.4, 120.1, 125.1, 126.8, 127.1, 127.3, 129.2, 129.4, 130.2, 130.6, 137.6, 138.6, 140.8, 141.7, 143.3, 143.7, 153.8, 167.8. Anal. Calcd for C_31_H_30_N_2_O_2_: C, 80.49; H, 6.54; N, 6.06, Found: C, 80.21; H, 6.74; N, 6.19.

2.2.9.N-[(cyclohexylcarbamoyl)(2-methoxyphenyl)methyl]-N-(9H-fluoren-2-yl)prop-2-ynamide (5i). White solid; yield (46%); m.p.137-139°C; FT-IR (KBr): 1628, 2101, 2854, 2931, 3085, 3281cm^-1^, ^1^H NMR (500 MHz, CDCl_3_) δ ppm:1.01-2.01(m, 10H), 2.77(s, 1H), 3.73-3.94 (m,6H), 5.49 (bs,1H), 6.66-6.78 (m, 2H), 7.06-7.71 (m, 9H).^13^C NMR (125 MHz, CDCl_3_): δ = 24.7, 24.8, 25.4, 32.8, 32.8, 36.7, 48.8, 55.2, 58.9, 76.5, 80.1, 110.1, 119.1, 120.1, 120.3, 121.8, 125.0, 126.8, 126.9, 127.1, 128.9, 130.1, 131.1, 137.6, 140.8, 141.5, 143.0, 143.6, 153.8, 157.4, 168.2.

2.2.10.N-[(cyclohexylcarbamoyl)(3-methoxyphenyl)methyl]-N-(9H-fluoren-2-yl)-prop-2-ynamide (5j). White solid; yield 55 %; mp 146-148°C; FT-IR (KBr): 1643, 2106, 2851, 2932, 3054, 3282 cm ^-1^. ^1^H NMR (500 MHz, CDCl_3_): δ =1.04-1.99 (m, 10H), 2.80(s, 1H), 3.63 (s, 3H), 3.81-3.88 (m, 3H), 5.58 (bs,1H), 6.01 (s,1H), 6.77 (m, 3H), 7.12 (m, 2H), 7.27-7.4 (m,3H), 7.54 (d, ^3^*J*_HH_= 7.35, 1H), 7.6 (d, ^3^*J*_HH_= 7.9, 1H), 7.74 (d, ^3^*J*_HH_=7.5, 1H).^13^C NMR (125 MHz, CDCl_3_): δ =24.7, 24.8, 25.4, 32.7, 36.7, 48.8, 55.1, 65.1, 76.2, 80.6, 114.7, 115.4, 119.4, 120.1, 122.6, 125.1, 126.8, 127.1, 127.3, 129.3, 129.4, 135.1, 137.5, 140.7, 141.8, 143.3, 143.6, 153.9, 159.4, 167.5.

2.2.11.N-[(cyclohexylcarbamoyl)(4-methoxyphenyl)methyl]-N-(9H-fluoren-2-yl)-prop-2-ynamide (5k).White solid; yield 65 %; mp 164-165 °C; FT-IR (KBr): 1643, 2106, 2851, 2932,3282, 3222, 3055 cm ^-1^.^1^H NMR (500 MHz, CDCl_3_): δ =1.01-1.99 (m, 10H,), 2.79(s, 1H), 3.73(s, 3H), 3.82-3.89 (m, 3H), 5.55 (bs,1H), 6.05 (s,1H), 6.71-7.59 (m, 10H).^13^C NMR (125 MHz, CDCl_3_): δ =24.7, 24.8, 25.4, 32.8, 36.7, 48.8, 55.1, 64.3, 76.2, 80.4, 113.8, 119.4, 120.1, 125.1, 125.6, 126.8, 127.1, 127.4, 129.5, 131.7, 137.4, 140.7, 141.7, 143.3, 143.7, 153.8, 159.7, 167.9. Anal. Calcd for C_31_H_30_N_2_O_3_: C, 77.80; H, 6.32; N, 5.85, Found: C, 77.92; H, 6.36; N, 5.89.

2.2.12.N-[(cyclohexylcarbamoyl)(3-fluorophenyl)methyl]-N-(9H-fluoren-2-yl)-3-phenylprop-2-ynamide (5l).white solid; yield 50 %; mp 210-211 °C; FT-IR (KBr): 1693, 2218, 2854, 2931, 3061, 3321cm ^-1^.^1^H NMR (500 MHz, CDCl_3_): δ = 1.09-2.03(m, 10H), 3.79-3.91(m, 3H), 5.86(bs,1H), 6.08(s, 1H), 6.74-6.97(m, 3H), 7.01-7.06(m, 4H), 7.12-7.27(m, 11H).^13^C NMR (125 MHz, CDCl_3_): δ =24.7, 24.8, 25.4, 32.7, 32.8, 36.8, 48.9, 64.7, 82.4, 92.5, 115.5, 115.7, 117.1, 117.3, 119.5, 120.2, 125.1, 125.9, 126.8, 127.1, 128.2, 129.3, 129.9, 132.4, 136.4, 138.1, 140.7, 141.8, 143.5, 143.7, 155.2, 162.4(d, ^1^*J*_CF_ = 245.7 Hz, CF), 167.5. Anal. Calcd for C_36_H_31_FN_2_O_2_: C, 79.68; H, 5.76; N, 5.16, Found: C, 79.83; H, 5.54; N, 5.21.

2.2.13.N-[(3-chlorophenyl)(cyclohexylcarbamoyl)methyl]-N-(9H-fluoren-2-yl)-3-phenylprop-2-ynamide (5m).white solid; yield 60 %; mp191-192 °C; FT-IR (KBr): 1655, 2216, 2854, 2935, 3082, 3264 cm^-1^. ^1^H NMR (500 MHz, CDCl_3_): δ = 1.12 -2.03 (m, 10H), 3.91-3.8(m, 3H), 5.86(bs,1H), 6.07(s,1H), 7.01-7.15(m, 6H), 7.22-7.42(m, 7H), 7.56(d, ^3^*J*_HH_= 7.4, 1H), 7.66(d, ^3^*J*_HH_= 8.0, 1H), 7.79(d, ^3^*J*_HH_= 7.5,1H).^13^C NMR (125 MHz, CDCl_3_): δ = 24.7, 24.8, 25.4, 32.7, 32.8, 36.8, 48.9, 64.6, 82.4, 92.6, 119.5, 120.1, 120.2, 125.1, 126.8, 127.1, 127.2, 128.2, 128.3, 128.7, 129.3, 129.6, 129.9, 130.4, 132.4, 134.2, 136.0, 137.9, 140.7, 141.8, 143.5, 143.7, 155.2, 167.4.

2.2.14.N-[(3-bromophenyl)(cyclohexylcarbamoyl) methyl]-N-(9H-fluoren-2-yl)-3-Phenylprop-2- ynamide (5n). White solid; yield 63 %; mp 189-190 °C; FT-IR (KBr): 1655, 2215, 2854, 2934, 3082, 3267cm ^-1^. ^1^H NMR (500 MHz, CDCl_3_): δ = 1.12-2.03(m, 10H), 3.80-3.91(m, 3H), 5.86(bs,1H), 6.07(s,1H), 7.01-7.48(m, 16H), 7.56 (d, ^3^*J*_HH_= 7.4Hz, 1H), 7.67(d, ^3^*J*_HH_= 8.0 Hz, 1H), 7.79(d, ^3^*J*_HH_= 7.5Hz,1H).^13^C NMR (125 MHz, CDCl_3_): δ = 24.7, 24.8, 25.4, 32.7, 32.8, 36.8, 48.9, 64.5, 82.3, 92.6, 119.5, 120.1, 120.2, 122.3, 125.1, 126.8, 127.1, 127.2, 128.2, 128.8, 129.4, 129.8, 129.9, 131.7, 132.4,133.3,136.2, 137.8,140.7, 141.8, 143.5, 143.7, 155.2,167.4. 

2.2.15.N-[(4-chlorophenyl)(cyclohexylcarbamoyl) methyl]-N-(9H-fluoren-2-yl)- 3- Phenylprop-2-ynamide (5o). White solid; yield 76%; mp 200-204 °C; FT-IR (KBr): 1655, 2216, 2932, 2854, 3082, 3271cm^-1^.^1^H NMR (500 MHz, CDCl_3_): δ= 1.12-2.0 (m, 10H),3.8-3.9(m,3H),5.82(s,1H),6.1(s,1H), 7.00-7.02(m,3H), 7.13(t, 2H),7.2(m, 4H), 7.27-7.24(m, 1H), 7.36-7.33(m, 1H), 7.41(t, 1H),7.57(d, J=7.3, 1H),7.6d, J=8.0, 1H), 7.79(d, J= 7.5, 1H).^13^C NMR (125 MHz, CDCl_3_): δ= 24.7, 25.4, 29.6, 32.7, 32.8, 36.8, 48.9, 64.2, 82.4, 92.6, 119.5, 120.1, 120.2,125.1, 126.8,126.9,127.1,127.2,128.2,128.6,129.4,129.9, 131.7,131.8,132.4,132.5, 132.6, 134.6, 137.2,137.8,140.7,141.8,143.5,143.7, 155.2,167.6.

2.2.16.N-[(4-bromophenyl)(cyclohexylcarbamoyl)methyl]-N-(9H-fluoren-2-yl)-3- Phenylprop-2- ynamide (5p).White solid; yield 85%; mp 209-210 °C; FT-IR (KBr): 1656, 2216, 2931, 2853, 3079, 3277 cm^-1^.^1^H NMR (CDCl_3_): δ = 2.06-1.08(m, 10H),3.91-3.80(m, 3H), 5.81(d,1H), 6.08(s, 1H),7.02-7.(m,2H),7.15-7.12(m, 5H), 7.27-7.24 (m, 2H), 7.36-7.34(m, 3H), 7.41(t,1H), 7.57(d, J=7.4, 1H), 7.66 (d, J=8.0, 1H), 7.79 (d, J= 7.5, 1H),7.35 (m, 3H).^13^C NMR (125 MHz, CDCl_3_): δ = 24.7, 24.8, 25.4, 29.6, 32.7, 32.8, 36.8, 48.9, 64.3, 82.3, 92.6, 119.5, 120.1, 120.2, 122.9, 125.1,126.8, 127.1, 127.2, 128.2, 129.4, 129.9, 131.6,131.9, 132.4,133.0, 137.8, 140.7,141.8, 143.5,143.7,155.2, 167.6. Anal. Calcd for C_36_H_31_BrN_2_O_2_: C, 71.64; H, 5.18; N, 4.64, Found: C, 70.87; H, 5.27; N, 4.54.

2.2.17.N-[(cyclohexylcarbamoyl)(2-nitrophenyl)methyl]-N-(9H-fluoren-2-yl)-3- Phenylprop-2-ynamide (5q). Pale yellowish solid; yield 60%; mp 152-154 °C; FT-IR (KBr): 1684, 2214,2930, 2853,3066, 3262 cm^-1^.^1^H NMR (500 MHz, DMSO-d6):δ= 1.72-0.94(m, 10H), 3.59-3.57(m,1H),3.82-3.73(m,2H), 7.07-7.05(m,2H), 7.45-7.21(m, 10H),7.55(d, J= 7.4, 1H), 7.77-7.64(m, 2H), 7.83(d, J= 7.4, 1H),8-7.98(m,1H), 8.25(d, J= 7.6, 1H). ^13^C NMR (125 MHz, CDCl_3_): δ = 24.2, 25.1,31.9, 36.2, 39.0,48.1,59.7,82.8, 90.5, 119.4,120.3, 125.0, 127.0, 128.8,129.6, 130.4, 131.7,132.5, 125.0, 127.0, 128.8, 129.6, 130.4, 131.7, 132.5, 133.2, 137.4, 140.1, 140.9, 143.4, 149.1, 153.1, 167.1.

2.2.18.N-[(cyclohexylcarbamoyl)(3-nitrophenyl)methyl]-N-(9H-fluoren-2-yl)-3- Phenylprop-2-ynamide (5r). White solid; yield 83 %; mp 189-190 °C; FT-IR (KBr): 1655, 2217, 2935, 2853,3081, 3260 cm ^-1^.^1^H NMR (500 MHz, CDCl_3_): δ= 2.07-1.20 (m, 10H), 3.91-3.79(m, 3H), 6.24(m, 2H), 7.02-7.67(m,13H), 7.78(d,J=7.5,1H),8.09-8.11(m,1H), 8.23-8.24(m,1H).^13^C NMR (125 MHz, CDCl_3_): δ = 24.7, 25.4, 29.7,32.7,32.8,36.8, 49.0,63.9,82.1, 93.2, 119.8,120.2, 122.4, 123.5, 124.6, 125.1, 125.3, 126.9, 127.0, 127.3, 128.3, 129.1, 129.2, 130.2, 130.8, 132.3, 135.9, 136.3, 137.3, 140.4, 142.1, 143.7, 143.8, 147.9, 155.4, 167.1.

2.2.19.N-[(cyclohexylcarbamoyl)(4-nitrophenyl)methyl]-N-(9H-fluoren-2-yl)-3- phenylprop-2-ynamide (5s). White solid; yield (50%); mp 200-203°C; FT-IR (KBr): 1631, 2200, 2924, 2857,3339 cm ^-1^.^1^H NMR (500 MHz, CDCl_3_): δ= 1.26- 2.10 (m, 10H), 3.88- 3.94 (m, 3H, CH_2_),6.10 (bs, 1H),6.21(s, 1H),8.13-7.05 (m, 16H).^ 13^C NMR (125 MHz, CDCl_3_): δ = 22.7, 24.7, 25.4, 29.3, 31.4, 31.9, 36.8, 49.0, 64.2, 83.2, 94.5, 119.8, 120.4, 123.4, 123.9, 124.4, 125.2, 126.9, 127.0,127.4, 128.3, 129.1,130.2, 131.2, 132.5,140.5, 141.0, 143.7, 143.8,167.1. Anal. Calcd for C_36_H_31_N_3_O_4_: C, 75.90; H, 5.49; N, 7.38, Found: C, 74.85; H, 5.54; N, 7.43.

2.2.20.N-[(cyclohexylcarbamoyl)(4-methylphenyl)methyl]-N-(9H-fluoren-2-yl)-3-Phenylprop-2-ynamide(5t).White solid; yield 62 %; mp 165-167 °C; FT-IR (KBr):1642, 2219, 2855, 2926 and, 3064, 3257cm ^-1^.^1^H NMR (500 MHz, CDCl_3_): δ= 1.04-2.00(m, 10H), 2.28(s,3H), 3.84(m,3H),5.66(d, 1H),6.09(s,1H),7-7.41(m,13H),7.55(d, J=7.6, 1H),7.63(d, J=8.0, 1H),7.77(d, J=7.5, 1H).^13^C NMR (125 MHz, CDCl_3_): δ = 20.9, 21.1,24.7,24.8, 25.47,32.8,36.8,48.8, 65.0, 82.6, 92.1,119.3,119.8,120.1, 120.3, 124.8, 125.1, 126.8, 126.9, 127.3, 128.2, 129.2,129.5,129.8,129.9,130.2, 130.9, 132.4, 138.3, 138.4,140.9, 141.4, 143.2, 143.3, 143.7, 155.1, 168.1. Anal. Calcd for C_37_H_34_N_2_O_2_: C, 82.50; H, 6.36; N, 5.20, Found: C, 82.32; H, 6.57; N, 5.48.

2.2.21.N-[(cyclohexylcarbamoyl)(2-methoxyphenyl)methyl]-N-(9H-fluoren-2-yl)-3-phenylprop-2-ynamide (5u). White solid; yield 65%; mp 186-188 °C; FT-IR (KBr): 1685, 2213, 2858, 2931, 3067,3335cm^-1^.^1^H NMR (500 MHz, CDCl_3_): δ= 1.04-2.04(m, 10H), 3.75- 3.91(m, 6H),5.58(d,1H), 6.45(s, 1H),6.70(s, 1H), 6.78(d,1H), 7.02(m,2H), 7.39-7.11 (m,9H),7.53(d, 1H),7.57(d,1H), 7.74 (d, 1H). ^13^C NMR (125 MHz, CDCl_3_): δ = 24.8,25.5,29.6, 31.2, 32.8, 36.7, 48.7, 55.2, 59.1, 82.9, 91.6,110.1,118.9, 120.1, 120.3, 120.5,122.2,125.1, 126.7, 126.8, 127.2, 128.1,129.1, 129.6, 130.1,131.1,138.4,132.3, 141.0,143.0, 141.2, 143.7,155.1,157.4, 168.5.

2.2.22.N-[(cyclohexylcarbamoyl)(3-methoxyphenyl)methyl]-N-(9H-fluoren-2-yl)-3-Phenylprop-2-ynamide (5v). White solid; yield 85 %; mp 187-189 °C; FT-IR (KBr): 1655, 2217, 2854, 2932, 3089,3265 cm^-1^.^1^H NMR (500 MHz, CDCl_3_): δ= 1.15-2.01(m, 10H), 3.65 (m, 3H),3.84(m,3H),5.78(bs, 1H, NH),6.80(m, 3H), 6.1(s, 1H), 7.01(m,2H), 7.14(m, 3H),7.25(m, 2H), 7.33(m, 1H), 7.4(t, 1H), 7.55(m, 2H), 7.64(d,1H),7.77(d, J=7.5, 1H). ^13^C NMR (125 MHz, CDCl_3_): δ = 24.7, 24.8, 25.4, 32.7, 36.7, 48.8,55.1,65.2,82.6, 92.2, 114.6, 115.4, 119.3, 120.1, 120.2,122.5, 125.1,126.8,126.9,127.3,128.2,129.4,129.5, 129.8, 132.3, 135.4, 138.3, 140.8,141.5, 143.2, 143.7, 155.1, 159.4, 167.8. Anal. Calcd for C_37_H_34_N_2_O_3_: C, 80.12; H, 6.18; N, 5.05, Found: C, 81.11; H, 6.24; N, 5.09.

2.2.23.N-[(cyclohexylcarbamoyl)(4-methoxyphenyl)methyl]-N-(9H-fluoren-2-yl)-3-Phenylprop-2-ynamide (5w). White solid; yield 50 %; mp 169-171 °C; FT-IR (KBr): 1642, 2217, 2854, 2932,3074, 3266 cm ^-1^. ^1^H NMR (500 MHz, CDCl_3_): δ = 1.05-2.01 (m, 10H), 3.74(s, 3H), 3.78-3.90(m,3H), 5.7 (bs,1H), 6.12(S,1H), 6.73(d,2H), 7.10-7.42(m, 9H), 7.01(d, 2H),7.55 (d, ^3^*J*=7.4, 1H), 7.63 (d, J=7.9, 1H),7.77( d, J=7.5, 1H).^13^C NMR (125 MHz, CDCl_3_): δ = 24.7, 24.7, 25.4, 32.8, 36.7, 48.8, 55.1, 64.3, 76.2, 80.4, 113, 119.4, 120.1, 125.1, 125.6, 126.8, 127.1, 127.4, 129.5, 131.7, 137.4, 140.8, 141.7, 143.3, 143.7, 153.8,159.7,167.9. Anal. Calcd for C_37_H_34_N_2_O_3_: C, 80.12; H, 6.18; N, 5.05, Found: C, 79.17; H, 6.29; N, 5.20.

*In-vitro evaluation of anti-mycobacterial activity*

*In vitro* anti-mycobacterial activity evaluations of the compounds were done by the broth dilution method) against BCG (1173P2) in addition to several sensitive and resistant strains of *M.* *tuberculosis* (H37Rv, IHMT149/09, HPV115/08 and HPV65/08) and ethambutol were used as standard controls.

The test compounds were initially dissolved in DMSO to give a concentration of 1 or 2 mg/L. All wells of micro plates received 100 µL of freshly prepared Middle broke 7H9 medium (Himedia, India), except first column. 200 µL of distilled water was added to the first column of 96 well plates to minimize evaporation of the medium in the test wells during incubation. Then 100 µL of test compounds with desired concentrations (2000 µL) were added to the wells of the first row (each concentration was assayed in duplicate) and serial dilution was made from the first row to the last. 

Microbial suspension of BCG (1173P2) (100 µL), which had been prepared with standard concentration of 0.5 Mcfarland and diluted with 1:10 proportion by the distilled water, was added to all test wells. Plates were then sealed and incubated for 4 days at 37 °C. After that 12 µL Tween 80 10% and 20µL Alamar blue 0.01% (Himedia, India) were added to each test well.The plates were re-incubated at 37 °C.The results were assessed after 24 and 48 h. A blue color was interpreted as no bacterial growth, and color change to pink was scored as bacterial growth. Wells with a well-defined pink color were scored as positive for growth. The MIC (minimal inhibition concentration) was defined as the lowest drug concentration, which prevented a color change from blue to pink. Ethambutol (Irandaru, Tehran) were used as positive control and DMSO as negative control.

*Brine shrimp toxicity study*

Brine shrimp lethality bioassay (28-31) was carried out to explore the toxicity of selected compounds with anti-mycobacterial potency. 

Dried cysts (1 g cyst per liter) of brine shrimp (*Artemia salina*) were hatched in a bottle containing artificial sea water (3.5% (w/v) marine salts/distilled water) at 28–30 °C with strong aeration (flow rate of 7 L/min), under a continuous light regime (1600 lux) for 30-35 h. Consequently, the newly hatched brine shrimp larvae (nauplii) were separated from the remaining cysts and collected with a pipette from the lighted side and concentrated in Petri dishes to be immediately utilized for bioassay. Assays were carried out in 24-well flat test plates (Orange Scientifique, Belgium). 

Acetone100% (Merck, Germany) was utilized for the preparation of different concentrations (1000, 100, 10 and 1 μg/mL) of tested compounds, in triplicates. Each well of treated groups exposed with several concentration of acetone dissolved compounds in the basic salt medium (3.5% (w/v) marine salts /distilled water in addition to poly ethylene glycol (PEG) 6000 (Merck, Germany)1.2%, while control groups only received basic salt medium. Gallic acid (Merck, Germany) was utilized as positive control; respectively. Following evaporation of vehicle solvent, entire wells introduced with 10 fresh nauplii and put on a shaker with 40 rpm to be aerated at room temperature. After 24 h, the numbers of survivors (larvae were considered dead if they did not exhibit any internal or external movement during several seconds of observation) were counted by microscope AC 230V, 50 Hz (Sairan, Iran) and recorded to determine the corrected mortality via following formula: 

Corrected mortality (%) = [(Mm_ct_)_t_ - (Mm_ct_)_c _/ 100 - (Mm_ct_)_c_] * 100

Here: 

Mm_ct _(mortality of individuals at time t %) = [N_Mm_ (number of died individuals) / N_0_ (initial number of living individuals in every test well at the beginning of the test)] * 100 

(Mm_ct_)_t _= calculated Mm_ct _for treated test wells

(Mm_ct_)_c _= calculated Mm_ct _for control test wells

On the subject of calculated corrected mortality, relevant 50% lethality doses (LD_50_)s with 95% confidence intervals were estimated by GraphPad Prism 5.0 (2007) for each selected anti-mycobacterial compound (30). 

## Results and Discussion

In order to synthesize fluorene bisamide derivatives, 2-fluoreneamine 1, aromatic aldehydes2, carboxylic acids 3 and cyclohexylisocyanide4 were treated in methanol at room temperature as a modal reaction. After completion of the reaction, the purified products were characterized by IR, ^1^H, and ^13^C NMR spectra. For example, the IR spectrum of 5b showed absorptions at 3281(N-H), 2111 (C≡C), and 1677 (C=O) cm^-1^, indicating the presence of these functional groups in the proposed structure. The ^1^H NMR spectrum of 5b exhibited a multiplet at δ = 1.17-1.68ppm for 5CH_2_ group Of cyclohexyl ring, a singlet for C≡CH group at δ= 2.82 ppm, two other singlets for NH and N-CH at δ = 5.72 and 6.01 ppm, respectively; also a multiplte for three protons of C*H*_2_ and NH-C*H *at δ = 3.78-3.90, finally a multiplet at δ = 7.06-7.76 ppm for 11 aromatic protons were appeared. The ^1^H NMR spectra of the related compounds 5 are similar to those of 5b, except for the substitutes, which exhibit characteristic signals with appropriate chemical shifts. The ^1^H decoupled ^13^C NMR spectrum of 5b showed 28 resonances.

To explore the scope and limitations of this versatile reaction, we have examined various aromatic aldehydes with 2-fluorenamine, and cyclohexyl isocyanide in the presence of acetylene carboxylic acid or phenyl acetylene carboxylic acid in methanol at room temperature. As indicated Table 1, the reactions proceed efficiently and led to formation of fluorene bisamide derivatives 5a-k and 5l-w in good yields, respectively. All the synthesized compounds were evaluated for anti-mycobacterial activity and the results are summarized in Table 1.

As can be seen from Table 1, in compounds 5, the nature of R^1^and X and also the location of X functional group on aromatic ring were investigated on *Mycobacterium bovis *activity. In compounds 5a-c and 5d,e changing of halogen from F to Br in position 3 and 4 has relatively same effect (3.9 and 3.12 μg/mL). In compounds 5f,g in which X is NO_2_ (very strong electron-withdrawing group), different location has different effect; in this case *ortho* isomer of these compounds is characterized by higher anti-bacterial activity than *meta* isomer (3.62 *vs *15.62 μg/mL).

About electron-donating substitution (Me or OMe) at positions 2 or 3 or 4, Me in *para* position relatively has more activity than OMe in different position.

It is important to note that when phenyl acetylene carboxylic acid was used instead of acetylene carboxylic acid in scheme 1 in the same reaction conditions, the obtained products, 5l-w, have poor activity against *Mycobacterium bovis *in contrast to products 5a-l. The obtained results show substitution on triple bond is important and H more active than Ph on triple bond.

It would be no doubt that investigating cytotoxicity properties of certain drug candidates is one of the key parameters affecting their fate in lead identification and following that further phases during drug discovery procedures. Recently, a number of toxicity tests have been developed in which the response has been demonstrated in invertebrates. The brine shrimp lethality bioassay (28-31) as one of these assays have the virtue of being economical, reproducible, easy to handle, and environmentally relevant deliberated a practical method for preliminary assessment of toxicity. If given factors as temperature, composition and salinity of the medium and the age of the larvae are considered, a fairly satisfactory repeat-ability is attained. Though, the brine shrimp assay is rather inadequate as regards the elucidation of the mechanism of action,(31) it is very practical to assess the toxicity of the natural and synthetic lead compounds. Numerous investigations during recent decades have investigated that the nature of the systems in brine shrimp which respond to drugs appears to be similar to those in mammals that has ended up with proposing this bioassay for screening biological activities.

In current study, the acute toxicity of anti-mycobacterial compounds, three of them with uppermost anti-mycobacterial potency were tested by means of the *A. salina* short-term bioassay at Pasteur Institute (Tehran, Iran). 

The related LD_50_s demonstrated based on analytical method non-linear regression (dose response inhabitation). Analyzed data showed selected derivatives exposed 50% lethality to nauplii table 2. in doses between 6.06 to 102.7 µg/mL. 

To discover the selectivity of anti-mycobacterial property for each tested compounds, a selectivity index (SI) was calculated by dividing LD_50 _to MIC that the range of calculated SIs was from 0.77 to 43.88. There would be no doubt that considering SIs for syntactic compounds may lead to introducing a set of novel anti-mycobacterial candidates with minor side effects for further investigations. 

## Conclusion

In conclusion, the one-pot procedure for the efficient synthesis of chemical library of fluorene bisamide derivatives via multi-component condensation reaction was carried out. The structures of the products 5a-w were deduced from their IR, ^1^H NMR, and ^13^C NMR spectra. Elemental analyses (CHN) for novel compounds (5a, 5d, 5f, 5h, 5k, 5l, 5p, 5s, 5t, 5v, 5w) were done too. All compounds were evaluated for anti-mycobacterial activity. The synthesized compounds exhibited promising anti-tubercular activities against of *M. bovis*. Among 23 synthesized fluorine bisamide derivatives (5a-w), compound 5a has highest activity against *M. bovis, *additionally; compound 5k showed greatest potency against sensitive and resistant strains of *M.* *tuberculosis* (H37Rv, IHMT149/09, HPV115/08 and HPV65/08). Finally, these results make novel fluorene bisamide derivatives, alone or in combination of other agents, interesting molecule for more synthetic, and biological evaluation.
